# The Association Between Notching of the Right Ventricular Outflow Tract Flow Velocity Doppler Envelope and Impaired Right Ventricular Function After Acute High-Altitude Exposure

**DOI:** 10.3389/fphys.2021.639761

**Published:** 2021-04-01

**Authors:** Fangzhengyuan Yuan, Chuan Liu, Shiyong Yu, Shizhu Bian, Jie Yang, Xiaohan Ding, Jihang Zhang, Hu Tan, Jingbin Ke, Yuanqi Yang, Chunyan He, Chen Zhang, Rongsheng Rao, Zhaojun Liu, Jun Yang, Lan Huang

**Affiliations:** ^1^Institute of Cardiovascular Diseases of PLA, The Second Affiliated Hospital, Third Military Medical University (Army Medical University), Chongqing, China; ^2^Department of Cardiology, The Second Affiliated Hospital, Third Military Medical University (Army Medical University), Chongqing, China; ^3^Department of Geriatric Cardiology, Chinese PLA General Hospital, Beijing, China; ^4^Department of Medical Ultrasonics, The Second Affiliated Hospital, Third Military Medical University (Army Medical University), Chongqing, China

**Keywords:** high altitude, right ventricular function, right ventricular outflow tract, speckle tracking echocardiography, tissue Doppler imaging

## Abstract

**Introduction:**

Pulmonary artery pressure (PAP) is increased and right ventricular (RV) function is well preserved in healthy subjects upon exposure to high altitude (HA). An increase in PAP may trigger notching of the right ventricular outflow tract Doppler flow velocity envelope (RVOT notch), which is associated with impaired RV function in patients with pulmonary hypertension. However, whether HA exposure can induce RVOT notch formation and the subsequent impact on cardiac function in healthy subjects remains unclear.

**Methods:**

A total of 99 subjects (69 males and 30 females) with a median age of 25 years were enrolled in this study; they traveled from 500 to 4100 m by bus over a 2-day period. All subjects underwent a comprehensive physiological and echocardiographic examination 1 day before ascension at low altitude and 15 ± 3 h after arrival at HA. The RVOT notch was determined by the presence of a notched shape in the RVOT Doppler flow velocity envelope. The systolic PAP (SPAP) was calculated as Bernoulli equation SPAP = 4 × (maximum tricuspid regurgitation velocity)^2^+5 and mean PAP (mPAP) = 0.61 × SPAP+2. Cardiac output was calculated as stroke volume × heart rate. Pulmonary capillary wedge pressure (PCWP) was calculated as 1.9+1.24 × mitral E/e’. Pulmonary vascular resistance (PVR) was calculated as (mPAP-PCWP)/CO.

**Results:**

After HA exposure, 20 (20.2%) subjects had an RVOT notch [notch (+)], and 79 (79.8%) subjects did not have an RVOT notch [notch (−)]. In the multivariate logistic regression analysis, the SPAP, right ventricular global longitude strain (RV GLS), and tricuspid E/A were independently associated with the RVOT notch. The SPAP, mPAP, PVR, standard deviations of the times to peak systolic strain in the four mid-basal RV segments (RVSD4), peak velocity of the isovolumic contraction period (ICV), and the peak systolic velocity (s’) at the mitral/tricuspid annulus were increased in all subjects. Conversely, the pulse oxygen saturation (SpO_2_), RV GLS, and tricuspid annulus plane systolic excursion (TAPSE)/SPAP were decreased. However, the increases of SPAP, mPAP, PVR, and RVSD4 and the decreases of SpO_2_, RV GLS, and TAPSE/SPAP were more pronounced in the notch (+) group than in the notch (−) group. Additionally, increased tricuspid ICV and mitral/tricuspid s’ were found only in the notch (−) group.

**Conclusion:**

HA exposure-induced RVOT notch formation is associated with impaired RV function, including no increase in the tricuspid ICV or s’, reduction of RV deformation, deterioration in RV-pulmonary artery coupling, and RV intraventricular synchrony.

## Introduction

Travelers typically flock to high-altitude (HA) areas. However, hypobaric hypoxia at HA stresses the cardiopulmonary system (Parati et al., 2018). Due to HA-induced hypoxic pulmonary vasoconstriction (HPV), the pulmonary artery pressure (PAP) increases (Naeije, 2019), filling of the left ventricle and right ventricle decreases, and the left ventricular (LV)/right ventricular (RV) diastolic function is altered (Maufrais et al., 2017). In contrast, the LV/RV contractile function remains well-preserved (Osculati et al., 2016; Maufrais et al., 2019; Williams et al., 2019; Sareban et al., 2020). According to previous studies, mild pulmonary hypertension (PH) could decrease RV function, and the RV dysfunction progresses regardless of the treatment of lowering pulmonary vascular resistance (PVR) (van de Veerdonk et al., 2011; Huston et al., 2019).

Notching of the right ventricular outflow tract flow velocity Doppler envelope (RVOT notch) has been widely reported in patients with PH. The RVOT notch is caused by the reflected wave propagating to the pulmonary valve prior to the closure of the valve during a systole. The RVOT notch forms in the context of increased PAP and PVR, decreased pulmonary vascular compliance, and the presence of a reflecting site close enough to the pulmonary valve to allow for the velocity of the reflecting wave (Arkles et al., 2011; Ghimire et al., 2016).

The formation of the RVOT notch was mainly attributed to high PAP (Lopez-Candales and Edelman, 2012). As for patients with PH, it has been reported that the presence of an RVOT notch is associated with deteriorated RV function, such as reduced tricuspid annulus plane systolic excursion (TAPSE), RV fractional area change (FAC), peak systolic velocity of the tricuspid annulus (tricuspid s’), and the ratio of stroke volume index to RV end-diastolic area (Arkles et al., 2011; Lopez-Candales and Edelman, 2012). Even though PAP also increased in healthy subjects upon acute HA exposure, the amount of increase only ranged from mild to moderate. It is unclear whether this moderate increase of PAP induced by HPV led to RVOT notch formation. Additionally, since the RV function in healthy subjects upon acute HA exposure was reported to be well preserved; whether the presence of the RVOT notch in healthy subjects upon acute HA exposure is associated with deteriorated RV function is also unclear. Thus, the mechanism of RVOT notch formation and the relationship between RVOT notch and RV function at HA are worthy to investigate.

In a previous study, we had preliminarily reported that compared to the RVOT notch negative population, the correlation between PAP and intraventricular RV dyssynchrony was much greater in people with an RVOT notch. In this study, we tended to formally characterize the RVOT notch and comprehensively unravel the association between HA-induced RVOT notch and cardiac function.

## Materials and Methods

### Participants and Study Design

This study was approved by the Clinical Research Ethics Board of Army Medical University (Identification Code 201907501) and was registered at www.chictr.org.cn (ChiCTR-TRC-No.1900025728). In June 2019, we conducted this prospective cohort study on the Qinhai–Tibet Plateau. Han Chinese people who were born and permanently lived at a low altitude (≤500 m) were invited to participate in this study. A total of 111 participants were recruited at low altitudes (Chengdu, China, 500 m) to ascend to HA (Litang, China, 4100 m). Of all 111 subjects, five subjects withdrew from the study prior to ascending, and two subjects returned to low altitude because of severe HA illness. Ultimately, 104 subjects successfully ascended to 4100 m (Litang, China) by bus over a 2-day period. Due to the lack of suitable echocardiographic images in five subjects, the final analysis included 99 subjects. These subjects were healthy adults without HA exposure history in the past 6 months and had no recent medication use. Exclusion criteria included the presence of cardiopulmonary disease, cerebrovascular disease, liver disease, kidney disease, or malignant tumors. All physiological and transthoracic echocardiography examinations were performed 1 day prior to ascension at low altitude and 15 ± 3 h after arrived at HA. This study was conducted in accordance with the Declaration of Helsinki, and all subjects provided written informed consent.

### Assessment of Physiological Parameters

Pulse oxygen saturation (SpO_2_) was recorded using a pulse oximeter (ONYX OR9500, Nonin, Plymouth, MN, United States). Blood pressure was measured with the subject in a sitting position using an electronic sphygmomanometer (Omron HEM-6200, Japan) after a 10-minute rest period. Heart rate (HR) data were recorded using the synchronous electrocardiogram during echocardiography. We measured height and weight with a height and weight scale (RGZ-120, I WISH, China) 1 day before ascension at a low altitude. The body mass index (BMI) was calculated as weight/(height)^2^.

### Transthoracic Echocardiography

Echocardiographic examinations were performed by two experienced sonographers equipped with a 2.5 MHz adult transducer using a CX50 ultrasound system (Philips Ultrasound System, Andover, MA, United States) with the subject in the left lateral decubitus position. The dynamic echocardiographic images, which consisted of three consecutive cardiac cycles, were stored digitally for offline analysis using QLAB 10.5 (Philips Healthcare, Andover, MA, United States) in a blinded fashion. All examinations and measurements were performed according to the recommendations of the American Society of Echocardiography (Rudski et al., 2010; Mitchell et al., 2019), and the value of each echocardiographic parameter was averaged from measurements of three consecutive cardiac cycles.

LV end-diastolic volume, LV end-systolic volume, RV basal transverse diameter (RVD base), and RVD mid were measured. RV end-diastolic and end-systolic areas were determined using the apical four-chamber view by manually tracing the RV endocardium. TAPSE was recorded as the peak excursion of the lateral tricuspid annulus, which was measured with M-mode echocardiography. The maximum tricuspid regurgitation velocity (TRV) was evaluated using continuous-wave Doppler. The LV and RV inflow were measured using the apical four-chamber view of pulsed-wave Doppler. The maximum early (E) and late (A) diastolic velocities were recorded. A pulse tissue Doppler was used to assess the velocities of the mitral and tricuspid annuli on the lateral and septal aspects in early diastole and systole. The peak early diastolic velocity of the annulus (e’), peak systolic velocity of the annulus (s’), and peak velocity of the isovolumic contraction period (ICV) were recorded as the average of the lateral and septal values. Stroke volume (SV), left ventricular ejection fraction (LVEF), and cardiac output (CO) were calculated using LV volume data and HR (Lang et al., 2015). RV FAC was calculated as follows: FAC = (end-diastolic area - end-systolic area)/end-diastolic area × 100. Systolic pulmonary artery pressure (SPAP) was calculated with the following modified Bernoulli equation: SPAP = 4 × (TRV)^2^+5 (Yock and Popp, 1984). The mean PAP (mPAP) was calculated as follows: mPAP = 0.61 × SPAP+2 (Bossone et al., 2013). Pulmonary capillary wedge pressure (PCWP) was calculated using the following equation: PCWP = 1.9+1.24 × mitral E/e’ (Bossone et al., 2013). PVR was derived according to the equation PVR = (mPAP-PCWP)/CO (Bossone et al., 2013).

To investigate the RVOT blood flow, the pulse Doppler sample volume probe was placed 0.5–1 cm proximally to the pulmonic valve in the parasternal short-axis view. The RVOT acceleration time (AT_*RVOT*_) and RVOT ejection time (ET_*RVOT*_) were measured. The shapes of the RVOT Doppler flow velocity envelope were categorized as notch (+) (in the presence of a notching pattern) and notch (−) (when no notching pattern was present), as previously described (Arkles et al., 2011). The specific notch relevant parameter mid-systolic flow deceleration time (mid-systolic DT) was measured (Takahama et al., 2017).

### 2D-STE Analysis

Standard 2D grayscale images (frame rate > 60 fps) in the apical two-, three-, and four-chamber views were used for the LV and RV speckle tracking analyses. Apical, basal, and mid-level parasternal short-axis views of three consecutive beats were also recorded. Using QLAB software v10.5, the operator manually adjusted the region of interest to include all segments of the ventricular myocardium. These were then traced by an algorithm, which automatically calculated the peak strain of each segment on different images.

The LV global longitudinal strain (GLS) was defined as the mean of 17 segments that was calculated on the apical two-, three- and four-chamber images. The LV global circumferential strain (GCS) was defined as the average peak strain in each segment of the basal, mid-, and apical levels using parasternal short-axis images. The RV GLS was defined as the mean of six segments on the RV-focused four-chamber image.

To analyze LV/RV dyssynchrony, the time from the onset of the QRS complexes to the peak strain of each segment on ECG was calculated and correlated to the R-R interval according to Bazett’s formula: corrected interval = measured time to peak strain/(RR interval)^1/2^. The standard deviation (SD) of the correlated interval for the 12 mid-basal LV segments was termed the TS-12SD (Zoroufian et al., 2014), whereas the SD for the four mid-basal RV segments was termed the RV-SD4 (Badagliacca et al., 2015).

### Reproducibility

To determine the intraobserver variability, data from 10 subjects at a low altitude and 10 subjects at a HA were randomly selected and evaluated twice by the same observer with a 1-month interval between evaluations. Interobserver variability was determined by comparing the evaluations conducted by two separate observers blinded to each other. The intra- and interobserver variabilities for the echocardiographic variables are presented in Supplementary Table S1.

### Statistical Analysis

All statistical analyses were performed using SPSS software (Version 22.0, IBM Corp., Armonk, NY, United States). Continuous variables are presented as mean ± standard error of mean if normally distributed or as median and interquartile range if not normally distributed, according to the Kolmogorov-Smirnov test. The paired t-test or Wilcoxon test was used to analyze the differences between paired variables at low altitude and HA, whereas the unpaired variables in the notch (−) and notch (+) groups at low altitude or HA and the unpaired variables in notch (+) subjects with mid-systolic DT < 120 ms and with mid-systolic DT > 120 ms were compared using the independent sample *t*-test or Mann-Whitney *U* test. Univariate logistic regression analysis was used to identify the relevant variables associated with RVOT notch. The variables with *P*-value < 0.05 were included in the multivariate logistic regression analysis. Category variables are presented as a number (percentage) and compared using the chi-square test. Statistical significance was set at *P* < 0.05.

## Results

### Subject Characteristics

**FIGURE 1:**
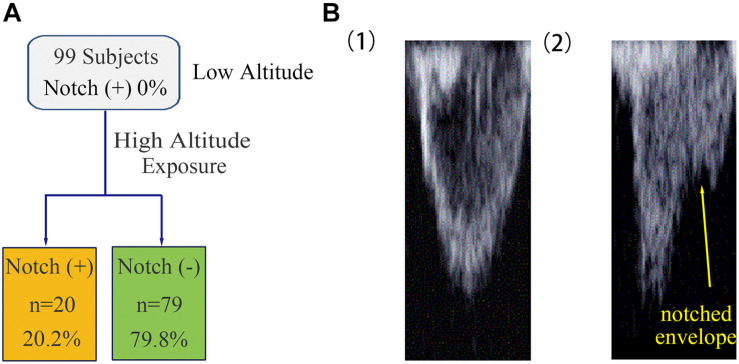
Occurrence of RVOT notch in healthy subjects at low altitude and upon acute HA exposure. **(A)** The occurrence of RVOT notch in healthy subjects was 0% at low altitude. Following HA exposure, the occurrence of RVOT notch in healthy subjects was 20.2%. **(B)** Morphology of the RVOT Doppler flow velocity envelope with or without RVOT notch. **(1)** RVOT Doppler flow velocity envelope in healthy subjects at low altitude was parabolic in shape. **(2)** Healthy subjects upon HA exposure with notched RVOT Doppler flow velocity envelope.

**FIGURE 2:**
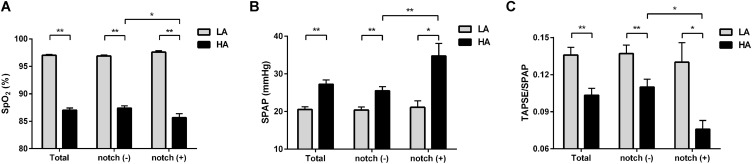
Differences in SpO_2_, SPAP, and TAPSE/SPAP between notch (−) and notch (+) groups at low altitude and HA. Different levels of **(A)** SpO_2_, **(B)** SPAP, and **(C)** TAPSE/SPAP in total subjects, notch (−) subjects and notch (+) subjects upon HA exposure are shown. Differences between notch (−) subjects and notch (+) subjects in **(A)** SpO_2_, **(B)** SPAP, and **(C)** TAPSE/SPAP were compared at low altitude and HA. Values are presented as mean and standard error of mean. *P < 0.05, **P < 0.01. Abbreviations as in Tables 1, 3.

The 99 subjects consisted of 69 men and 30 women with a median age of 25 years and an average BMI of 22.2 kg/m^2^. No subject was found to have an RVOT notch at low altitude. Upon HA exposure, an RVOT notch was identified in 20 subjects (20.2%) (Figure 1). There were no significant differences in age, sex, or BMI between the notch (+) and notch (−) groups. Following HA exposure, subjects in all groups had increased systolic blood pressure, diastolic blood pressure, and HR. Both groups also had decreased SpO_2_ following HA exposure, with the notch (+) group experiencing a lower SpO_2_ level than the notch (−) group (*P* = 0.039) (Table 1 and Figure 2).

**TABLE 1:** Physiologic parameters in the subjects with or without RVOT notch at low altitude and high altitude.

Baseline variables	Total (*n* = 99)			Notch (−) (*n* = 79)			Notch (+) (*n* = 20)		
			
	Low altitude	High altitude	*P-*value	Low altitude	High altitude	*P*-value	Low altitude	High altitude	*P*-value
Age, years	25.0 (21.3, 29.0)	−		25.0 (21.5, 28.5)	−		26.0 (21.0, 33.0)	−	
Male, *n* (%)	69 (69.7)	−		57 (72.2)	−		12 (60.0)	−	
BMI, kg/m^2^	22.2 ± 0.2	−		22.2 ± 0.3	−		22.4 ± 0.6	−	
HR, beats/min	63.0 (57.0, 72.5)	81.0 (73.5, 93.0)	** < 0.001**	62.0 (56.5, 72.5)	80.0 (72.5, 92.5)	** < 0.001**	66.0 (63.0, 75.8)	88.0 (79.3, 94.0)	** < 0.001**
SpO_2_, %	97.0 (96.0, 98.0)	88.0 (85.0, 90.0)	** < 0.001**	97.0 (96.0, 98.0)	88.0 (85.0, 90.0)	** < 0.001**	97.5 (97.0, 99.0)	86.0 (82.5, 88.0)**^∗^**	** < 0.001**
SBP, mmHg	117.0 (108.0, 126.0)	121.0 (111.0, 132.0)	**0.004**	117.0 (109.0, 126.0)	120.0 (111.0, 128.0)	**0.033**	122.0 (107.3, 134.3)	131.0 (113.0, 134.8)	**0.048**
DBP, mmHg	73.0 (66.0, 82.5)	81.0 (72.5, 90.5)	** < 0.001**	73.0 (64.5, 81.5)	79.0 (72.5, 89.5)	** < 0.001**	77.5 (67.5, 83.0)	85.0 (72.0, 96.0)	**0.032**

*BMI, body mass index; HR, heart rate; SpO_2_, pulse oxygen saturation; SBP, systolic blood pressure; DBP, diastolic blood pressure. Data are presented as mean ± standard error of the mean or median (25th to 75th quartile). ^∗^*P* < 0.05 compared with notch (−) at HA. The bolded values mean *p* value < 0.05.*

### LV Function

After HA exposure, SV and LV GLS decreased, GCS did not change, and the LVEF, CO, mitral ICV, and TS-12SD increased in all subjects. The mitral s’ in the notch (−) group increased upon HA exposure, but the mitral s’ in the notch (+) group did not change. Following HA exposure, the mitral E, mitral E/A, and mitral E/e’ decreased in all subjects. However, the mitral A increased in all subjects following HA exposure, with a higher value in the notch (+) group than in the notch (−) group. The mitral E/A at HA in the notch (+) group was lower than that in the notch (−) group (Table 2).

**TABLE 2:** Left ventricular parameters in subjects with or without RVOT notch at low altitude and high altitude.

Variables	Total (*n* = 99)			Notch (−) (*n* = 79)			Notch (+) (*n* = 20)		
	Low altitude	High altitude	*P*-value	Low altitude	High altitude	*P*-value	Low altitude	High altitude	*P*-value
**Conventional Doppler echocardiography**									
SV, ml	65.0 (57.2, 71.45)	57.5 (51., 66.35)	**< 0.001**	66.1 (57.2, 73.1)	58.5 (53.6, 66.5)	**< 0.001**	64.1 (56.9, 68.3)	55.7 (48.6, 66.4)	0.156
LVEF, %	59.2 (56.4, 61.5)	59.2 (55.8, 61.6)	0.841	58.5 (56.4, 61.5)	59.0 (56.2, 62.0)	0.784	60.0 (57.5, 62.3)	59.3 (54.8, 61.6)	0.458
CO, L/min	4.2 (3.5, 4.9)	4.6 (4.1, 5.5)	**< 0.001**	4.2 (3.5, 5.0)	4.6 (4.1, 5.4)	**< 0.001**	4.3 (3.5, 4.6)	4.7 (4.4, 6.1)	**0.008**
Mitral E, cm/s	93.4 (81.0, 110.4)	81.6 (71.9, 97.4)	**< 0.001**	93.4 (82.1, 111.0)	83.0 (72.6, 98.1)	**< 0.001**	92.7 (74.8, 107.6)	78.3 (64.9, 93.8)	**0.001**
Mitral A, cm/s	51.7 (44.4, 63.5)	59.4 (50.8, 68.4)	**< 0.001**	50.9 (44.3, 63.0)	57.2 (49.1, 67.0)	**< 0.001**	52.7 (48.8, 66.1)	64.5 (58.4, 73.7)^∗^	**0.007**
Mitral E/A ratio	1.7 (1.5, 2.1)	1.4 (1.2, 1.6)	**< 0.001**	1.7 (1.6, 2.1)	1.5 (1.2, 1.7)	**< 0.001**	1.6 (1.2, 2.2)	1.3 (1.1, 1.5)^∗^	**< 0.001**
**Pulse tissue Doppler imaging**									
Mitral s’, cm/s	10.5 (9.6, 11.6)	11.6 (10.3, 12.7)	**< 0.001**	10.5 (9.6, 11.7)	11.6 (10.3, 13.1)	**< 0.001**	10.6 (9.4, 11.5)	10.9 (9.6, 12.4)	0.297
Mitral e’, cm/s	15.1 (13.6, 16.3)	14.5 (13.4, 16.1)	0.663	15.1 (13.7, 16.2)	14.9 (13.4, 16.9)	0.346	14.1 (10.8, 16.7)	14.1 (13.4, 15.3)^∗^	0.701
Mitral E/e’ ratio	6.4 (5.7, 7.2)	5.6 (4.9, 6.4)	**< 0.001**	6.4 (5.6, 7.3)	5.5 (4.7, 6.4)	**< 0.001**	6.6 (5.9, 7.1)	5.7 (5.3, 6.5)	**0.006**
Mitral ICV, cm/s	8.6 ± 0.2	9.6 ± 0.2	**< 0.001**	8.7 ± 0.2	9.5 ± 0.2	**0.005**	8.5 ± 0.5	10.0 ± 0.6	**0.035**
**Speckle tracking imaging**									
LV GLS, %	−20.9 (−19.9, −21.7)	−19.1 (−17.8, −20.6)	**< 0.001**	−20.8 (−19.9, −21.6)	−19.2 (−18.1, −20.6)	**< 0.001**	−21.3 (−20.4, −22.3)	−18.7 (−17.3, −20.3)	**0.001**
LV GCS, %	−24.8 (−23.6, −26.7)	−24.6 (−23.3, −25.9)	0.118	−24.8 (−23.8, −26.9)	−24.6 (−23.6, −26.0)	0.106	−24.8 (−21.4, −26.7)	−23.6 (−22.6, −25.8)	0.520
TS-12SD, ms	19.7 ± 0.9	25.7 ± 1.0	**< 0.001**	20.1 ± 1.0	25.7 ± 1.2	**< 0.001**	18.3 ± 1.9	25.5 ± 2.2	**0.015**

SV, stroke volume; LVEF, left ventricular ejection fraction; CO, cardiac output; E, early diastolic transmitral or transtricuspid flow velocity; A, late diastolic transmitral or transtricuspid flow velocity; s’, mitral or tricuspid annulus systolic velocity; e’, mitral or tricuspid annulus early diastolic velocity; ICV, peak velocity of isovolumic contraction period at mitral or tricuspid annulus; LV, left ventricular; GLS, global longitude strain; GCS, global circumferential strain; TS-12SD, the standard deviation of time to peak systolic strain in 12 LV segments; Data are presented as mean ± standard error of the mean or median (25th to 75th quartile). *P < 0.05 compared with notch (−) at HA. The bolded values mean *p* value < 0.05.

### Pulmonary Hemodynamics and RV Function

**FIGURE 3:**
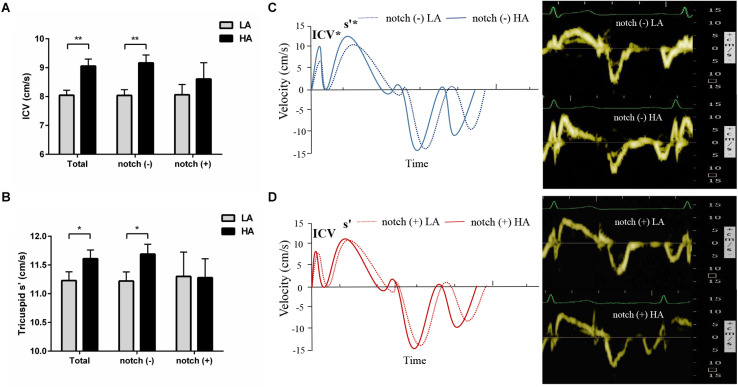
Tricuspid pulse Doppler velocity analysis in subjects with and without RVOT notch. Different changes in tricuspid ICV **(A)** and s’ **(B)** in notch (−) subjects and notch (+) subjects upon HA exposure. **(C)** and **(D)** Left panel: pulse tissue Doppler velocity curves of the tricuspid annulus in notch (−) and notch (+) subjects at low altitude and HA. Right panel: examples of the pulse TDI at tricuspid annulus in a subject without RVOT notch **(C)** and a subject with RVOT notch **(D)**. TDI, tissue Doppler imaging; *P < 0.05, **P < 0.01. Other abbreviations as in Table 3.

**FIGURE 4:**
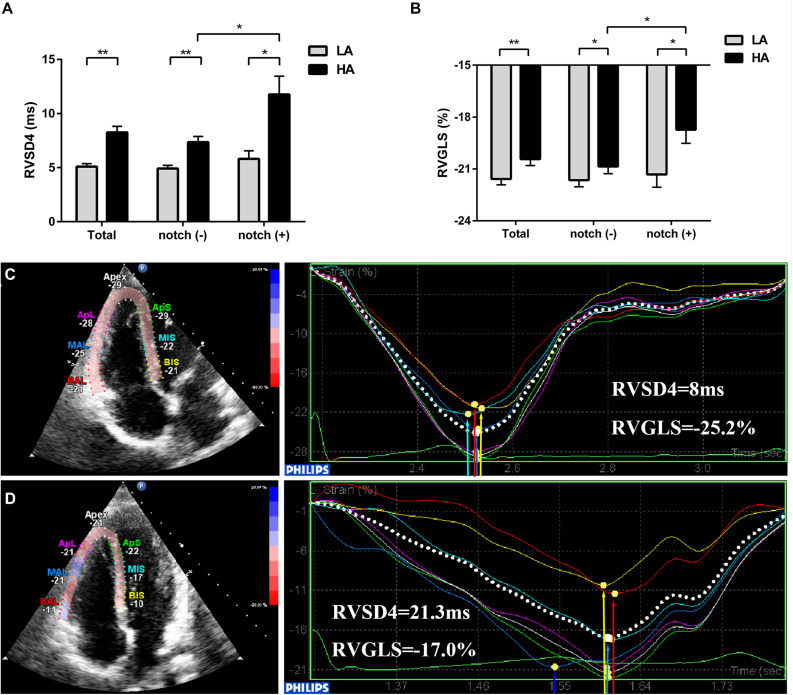
Evaluation of RV GLS and dyssynchrony in subjects with and without RVOT notch from low altitude to HA. Different levels of **(A)** RVSD4 and **(B)** RV GLS in total subjects, notch (−) subjects, and notch (+) subjects upon HA exposure are shown. **(C)** and **(D)** Examples illustrating the 2D-STE measurements of RV GLS and RV dyssynchrony in a subject without RVOT notch **(C)** and a subject with RVOT notch **(D)** at HA. *P < 0.05, **P < 0.01. Abbreviations as in Table 3.

Upon HA exposure, TRV, SPAP, mPAP, and PVR increased in all subjects; however, this increase was greater in notch (+) subjects. The AT_*RVOT*_, ET_*RVOT*_, AT_*RVOT*_/ET_*RVOT*,_ and PCWP decreased in all subjects, except for ET_*RVOT*_ in notch (+) subjects. The RVD base did not change in all subjects. The RVD mid increased in all subjects, which was majorly observed in the notch (+) group. The RV FAC, TAPSE, and RV GLS decreased in both groups, and the change in RV GLS was greater in the notch (+) group. The tricuspid s’ and tricuspid ICV increased after HA exposure in the notch (−) group (Figure 3). Additionally, the RV GLS decreased in both groups upon HA exposure and was lower in the notch (+) group than in the notch (−) group (Figure 4). The two-dimensional strain of the middle segment in the right ventricular free wall (2DS RVFW mid) and 2DS RVFW base in the notch (+) group at HA were lower than those in the notch (−) group. The TAPSE/SPAP decreased in all subjects after HA exposure, with a greater decrease in the notch (+) group (Figure 2). The RVSD4 increased in all subjects upon HA exposure, and it was more pronounced in the notch (+) group (Figure 4). After HA exposure, the tricuspid A in the notch (+) group was higher than that in the notch (−) group, and the tricuspid E/A in the notch (+) group was lower than that in the notch (−) group (Table 3). Categorizing notch (+) subjects into two groups according to mid-systolic DT (Takahama et al., 2017), we determined the RVD base and RVD mid were larger in mid-systolic DT < 120 ms group than those in mid-systolic DT > 120 ms group (Supplementary Table S2).

**TABLE 3:** Pulmonary circulation and right ventricular parameters in subjects with or without RVOT notch at low altitude and high altitude.

Variables	Total (*n* = 99)			Notch (-) (*n* = 79)			Notch (+) (*n* = 20)		
	Low altitude	High altitude	*P*-value	Low altitude	High altitude	*P*-value	Low altitude	High altitude	*P-*value
**Pulmonary hemodynamics**									
TR, *n* (%)	63 (63.6)	91 (91.9)	**< 0.001**	53 (67.1)	74 (93.7)	**< 0.001**	10 (50.0)	17 (85.0)	**0.018**
TRV, cm/s	196 (174, 220)	231 (202, 261)	**< 0.001**	195 (173, 220)	229 (194, 257)	**< 0.001**	209 (181, 226)	243 (222, 312)**	**0.020**
SPAP, mmHg	20.5 ± 0.7	27.2 ± 1.1	**< 0.001**	20.4 ± 0.8	25.5 ± 1.1	**< 0.001**	21.2 ± 1.7	34.8 ± 3.3**	**0.020**
mPAP, mmHg	14.4 (12.5, 16.9)	18.1 (15.0, 21.7)	**< 0.001**	14.3 (12.3, 16.9)	17.8 (14.2, 21.2)	**< 0.001**	15.7 (13.0, 17.6)	19.5 (17.0, 28.8)**	**0.020**
PVR, WU	1.2 (0.8, 1.8)	1.9 (1.3, 2.9)	**< 0.001**	1.2 (0.8, 1.7)	1.9 (1.3, 2.4)	**< 0.001**	1.3 (0.6, 1.8)	3.1 (1.4, 4.3)*	**0.037**
AT_*RVOT*_, ms	146.5 (135.1, 161.3)	116.0 (96.6, 134.4)	**< 0.001**	146.6 (135.1, 162.6)	118.1 (96.6, 134.1)	**< 0.001**	141.3 (131.8, 157.6)	112.9 (96.7, 137.6)	**0.002**
ET_*RVOT*_, ms	372.2 ± 35.96	360.0 ± 35.51	**0.001**	374.3 ± 33.8	361.7 ± 36.7	**0.002**	364.1 ± 43.5	353.4 ± 30.5	0.311
AT_*RVOT*_/ET_*RVOT*_	0.40 (0.35, 0.43)	0.32 (0.27, 0.38)	**< 0.001**	0.40 (0.35, 0.43)	0.32 (0.26, 0.37)	**< 0.001**	0.39 (0.36, 0.43)	0.33 (0.27, 0.40)	**0.002**
PCWP, mmHg	9.9 (8.9, 10.9)	8.9 (8.0, 9.8)	**< 0.001**	9.8 (8.9, 11.0)	8.7 (7.7, 9.8)	**< 0.001**	10.0 (9.2, 10.7)	9.0 (8.5, 10.0)	**0.006**
**Conventional Doppler echocardiography**									
RVD base, cm	3.5 ± 0.05	3.4 ± 0.06	0.378	3.6 ± 0.06	3.4 ± 0.07	0.055	3.3 ± 0.11^##^	3.5 ± 0.09	0.079
RVD mid, cm	3.2 ± 0.08	3.4 ± 0.08	**0.017**	3.2 ± 0.09	3.4 ± 0.09	0.119	3.0 ± 0.18	3.5 ± 0.14	**0.031**
RV EDA, cm^2^	20.2 (17.7, 23.0)	20.0 (17.3, 22.8)	0.380	20.4 (17.7, 24.2)	20.0 (17.7, 22.9)	0.129	19.3 (17.4, 21.1)	19.7 (17.1, 22.3)	0.458
RV ESA, cm^2^	10.9 (9.0, 12.5)	11.4 (9.8, 13.0)	0.065	11.2 (9.1, 12.9)	11.4 (9.8, 13.1)	0.273	9.8 (8.5, 11.8)	11.2 (9.6, 12.8)	0.065
RV FAC, %	46.6 ± 0.6	43.2 ± 0.5	**< 0.001**	46.5 ± 0.6	43.5 ± 0.5	**< 0.001**	47.1 ± 1.5	42.2 ± 1.1	**0.021**
TAPSE, mm	25.9 ± 0.4	23.8 ± 0.4	**< 0.001**	25.7 ± 0.4	24.1 ± 0.4	**0.003**	26.6 ± 0.9	22.9 ± 0.9	**0.002**
TAPSE/SPAP	1.2 (1.1, 1.5)	0.9 (0.7, 1.2)	**< 0.001**	1.2 (1.1, 1.5)	0.9 (0.8, 1.3)	**< 0.001**	1.1 (1.1, 1.4)	0.8 (0.5, 1.0)*	**0.023**
Tricuspid E, cm/s	66.6 ± 1.2	60.9 ± 1.5	**< 0.001**	67.0 ± 1.3	61.6 ± 1.6	**0.004**	65.1 ± 2.8	58.4 ± 3.7	**0.017**
Tricuspid A, cm/s	36.3 (30.5, 42.8)	42.1 (33.0, 52.5)	**< 0.001**	36.5 (30.6, 42.4)	39.7 (31.8, 48.5)	**< 0.001**	32.7 (29.3, 49.0)	51.5 (42.7, 57.7)*	**0.006**
Tricuspid E/A ratio	1.5 (1.8, 2.2)	1.5 (1.2, 1.7)	**< 0.001**	1.5 (1.8, 2.2)	1.5 (1.3, 1.7)	**< 0.001**	1.7 (1.4, 2.4)	1.2 (1.0, 1.6)**	**0.001**
**Pulse tissue Doppler imaging**									
Tricuspid s’, cm/s	11.2 ± 0.2	11.6 ± 0.2	**0.020**	11.2 ± 0.2	11.7 ± 0.2	**0.017**	11.3 ± 0.4	11.3 ± 0.3	0.662
Tricuspid e’, cm/s	14.3 ± 0.2	14.1 ± 0.3	0.211	14.5 ± 0.2	14.3 ± 0.3	0.403	13.7 ± 0.6	13.2 ± 0.7	0.229
Tricuspid E/e’ ratio	4.7 (4.1, 5.2)	4.5 (3.8, 4.9)	**0.046**	4.7 (4.1, 5.2)	4.4 (3.7, 4.9)	0.082	4.7 (4.3, 5.1)	4.6 (3.9, 5.0)	0.270
Tricuspid ICV, cm/s	7.9 (7.3, 8.8)	8.8 (7.3, 10.7)	**< 0.001**	7.9 (7.1, 8.7)	9.2 (7.3, 10.8)	**< 0.001**	7.8 (7.4, 9.1)	8.4 (6.7, 40.6)	0.341
**Speckle tracking imaging**									
RV GLS, %	−21.7 (−19.8, −23.9)	−20.1 (−17.7, −22.9)	**0.003**	−21.7 (−20.0, −24.2)	−20.5 (−18.6, −22.9)	**0.035**	−22.2 (−19.3, −23.3)	−18.4 (−16.0, −22.4)*	**0.015**
2DS RVFW apex, %	−23.6 ± 0.4	−21.9 ± 0.5	**0.005**	−23.5 ± 0.5	−22.3 ± 0.5	0.079	−23.9 ± 0.7	−20.3 ± 1.0	**0.004**
2DS RVFW mid, %	−23.3 ± 0.4	−22.2 ± 0.5	0.063	−23.5 ± 0.5	−22.8 ± 0.6	0.183	−22.6 ± 0.9	−20.0 ± 1.1*	0.057
2DS RVFW base, %	−18.0 (−15.0, −21.0)	−17.0 (−14.0, −20.0)	0.274	−18.0 (−15.0, −20.0)	−18.0 (−15.0, −20.0)	0.684	−18.0 (−14.3, −21.8)	−14.0 (−13.0, −19.0)*	0.093
RVSD4, ms	4.2 (3.1, 7.0)	6.6 (4.4, 11.6)	**< 0.001**	4.2 (3.1, 6.9)	6.4 (4.3, 9.8)	**< 0.001**	4.1 (3.4, 7.7)	12.1 (5.1, 18.0)*	**0.005**

*TR, tricuspid regurgitation; TRV, tricuspid regurgitation velocity; SPAP, systolic pulmonary artery pressure; mPAP, mean pulmonary artery pressure; PVR, pulmonary vascular resistance; AT_*RVOT*_, acceleration time of RVOT; ET_*RVOT*_, ejection time of RVOT; PCWP, pulmonary capillary wedge pressure; RV, right ventricular; EDA, end-diastolic area; ESA, end-systolic area; FAC, fraction area change; TAPSE, tricuspid annulus plane systolic excursion; RVD, right ventricular transverse diameter; 2DS RVFW, two-dimensional strain of right ventricular free wall. RVSD4, standard deviation of the time to peak systolic strain in the 4 mid-basal RV segments; Other abbreviation as in Table 2. Data are presented as mean ± standard error of the mean or median (25th to 75th quartile). ^##^*P* < 0.01 compared with notch (−) at low altitude, ^∗^*P* < 0.05, ^∗∗^*P* < 0.01 compared with notch (−) at HA. The bolded values mean *p* value < 0.05.*

### Determinants of RVOT Notch

In multivariate logistic regression analysis, SPAP (OR, 1.14; 95% CI, 1.05 to 1.23; *P* = 0.001), RV GLS (OR, 0.74; 95% CI, 0.60 to 0.93; *P* = 0.011), and tricuspid E/A (OR, 0.13; 95% CI, 0.03 to 0.68; *P* = 0.016) were found to be independently associated with RVOT notch (Supplementary Table S3).

## Discussion

Following rapid elevation gain, an RVOT notch was found in 20 (20.2%) healthy subjects. SpO_2_, RV deformation, the level of RV-PA coupling, and RV intraventricular synchrony were decreased in all subjects, while the RV afterload, biventricular s’, and ICV were increased in all subjects. However, the increase in the RV afterload and the decrease in SpO_2_, RV deformation, RV-PA coupling, and RV intraventricular synchrony were more pronounced in subjects with an RVOT notch. Moreover, the mitral s’, tricuspid s’, and tricuspid ICV in subjects with an RVOT notch did not increase upon HA exposure. These results suggest that HA exposure induced the RVOT notch formation, which may be related to impaired RV function.

### Association of RVOT Notch, PAP, and SpO_2_ Upon HA Exposure

In previous studies, the RVOT notch was mainly observed in patients with chronic PH, especially in those with pulmonary vascular disease (Arkles et al., 2011; Takahama et al., 2017). Patients with low arterial compliance, high arterial resistance, and/or a pulmonary embolism proximal to the pulmonary valve may have a pulmonary vascular reflected wave on Doppler, which is seen as a notched Doppler flow velocity contour in the RVOT if the reflected wave rapidly propagates to the pulmonary valve prior to its closure (Torbicki et al., 1999; Arkles et al., 2011). In this study, we found that RVOT notch formation in subjects upon HA exposure is associated with higher SPAP, which is in accordance with the previous finding in PH patients (Lopez-Candales and Edelman, 2012). Upon HA exposure, alveolar hypoxia-induced HPV and inhomogeneous pulmonary vasoconstriction facilitated the ventilation–perfusion matching in the regional lung (Sommer et al., 2008). According to previous studies, SpO_2_ decreases and SPAP increases with rising altitude (Penaloza and Arias-Stella, 2007). Furthermore, in patients receiving bosentan for PH, SpO_2_ levels significantly increase with an increase in altitude (Modesti et al., 2006; Mellor et al., 2014). Thus, patients with lower SpO_2_ are more likely to experience HPV of greater magnitude as an attempt to maintain the ventilation–perfusion balance, which leads to higher SPAP and PVR. Although the presence of an RVOT notch in patients with PH indicates a high SPAP (Kubba et al., 2016), the uneven HPV at HA might induce a reflection site proximal to the pulmonary valve, which leads to the formation of an RVOT notch.

### LV Function

Similar to previous studies, our results indicated that LV filling is decreased and LV contractile function is preserved in subjects upon HA exposure (Maufrais et al., 2017, 2019). Although the LV filling is decreased, the HR is increased. The maintained LV function might due to the increase of sympathetic activation. According to a previous study, LV function is not related to RVOT notch formation due to the absence of an RVOT notch in left-heart-disease-associated PH (Kushwaha et al., 2016). However, hypoxia may affect LV function in notch (+) subjects with a lower SpO_2_. Thus, the increase of mitral s’ in the notch (+) group was blunted in this study. At HA, no difference in TS-12SD was found between the two groups in this study, indicating that upon HA exposure, the SpO_2_ has little effect on LV dyssynchrony.

### Impact of RVOT Notch on RV Function Evaluated by Conventional Echocardiography

In our study, we found that the presence of an RVOT notch indicates impaired RV function in healthy subjects upon HA exposure, which is consistent with previous studies that reported advanced RV dysfunction in PH patients with RVOT notch (Arkles et al., 2011; Lopez-Candales and Edelman, 2012). Previous reports of the changes in RV FAC and TAPSE vary from a decrease (Kurdziel et al., 2017; Netzer et al., 2017), to no change (Maufrais et al., 2017, 2019), and to an increase upon HA exposure (Sareban et al., 2020). These inconsistent results may be due to different exposure times and ascending altitudes, or states of dehydration, all of which have been associated with RV adaptation upon HA exposure. In the present study, we found that the TAPSE and RV FAC decreased upon HA exposure. Although RV contractility may increase in response to the acutely increased RV afterload to maintain the pump function and RV-PA coupling (Naeije et al., 2014), the appropriately increased RV contractility upon HA exposure that is mirrored by load-dependent RV FAC and TAPSE may be under detected due to hypovolemia-induced RV FAC and TAPSE reduction via the Frank-Starling mechanism in patients with moderately increased PAP (Rudski et al., 2010; Najafian et al., 2015; Stembridge et al., 2016; Zhao et al., 2019).

TAPSE/SPAP is noninvasively measured for the evaluation of RV-PA coupling and correlates well with the gold standard multi-beat end-systolic/arterial elastance ratio in patients with PH (Tello et al., 2019; Richter et al., 2020). In this study, the decrease of TAPSE/SPAP in all subjects indicates that the level of RV-PA coupling was decreased in healthy subjects upon HA exposure. The more significant decrease of TAPSE/SPAP in the notch (+) group was attributed to the higher SPAP. The comparable TAPSE between notch (−) and notch (+) groups suggests that the RV contractility in the notch (+) group is maladaptive to the higher RV afterload upon HA exposure. TAPSE/SPAP has been reported to be positively correlated with VO_2_ peak and workload during cardiopulmonary exercise testing (Martens et al., 2018; Tello et al., 2018). Thus, although TAPSE/SPAP was maintained within a normal range, lower TAPSE/SPAP in the notch (+) subjects upon HA exposure may indicate lower work capacity.

### Impact of RVOT Notch on RV Function Evaluated by TDI

Upon HA exposure, the load-independent tricuspid s’ and ICV increased in the context of increased PAP (Abali et al., 2005; Rudski et al., 2010; Negishi et al., 2017), which indicates an increased RV inotropic function in response to the elevated RV afterload (Vonk Noordegraaf et al., 2017). This enhancement of myocardial contractility may be attributed to homeometric autoregulation or sympathetic activation (Rex et al., 2007, 2008). The association of RV contractility with sympathetic activation upon HA exposure is supported by a previous report, which suggests that the tricuspid s’ is correlated with HR (Sareban et al., 2020). However, in the notch (+) group, the tricuspid s’ and ICV did not increase to match the increased RV afterload, leading to a blunted RV contractile response upon HA exposure. This may be due to the fact that the positive inotropic effect of homeometric autoregulation or sympathetic activation was overwhelmed by the negative inotropic effect of hypoxia in notch (+) subjects with lower SpO_2_ (Silverman et al., 1997).

### Impact of RVOT Notch on RV Function Evaluated by 2D-STE

Although the conventional RV function parameters RV FAC and TAPSE were comparable in both groups at HA in this study, the 2D-STE RV function parameter RV GLS was lower in the notch (+) group, especially in the base and mid segments of RVFW. This may be due to the fact that the RV GLS is a more sensitive and accurate assessment for detecting subclinical RV dysfunction than TAPSE and RV FAC (Giusca et al., 2010; Li et al., 2018; Tamulenaite et al., 2018). Even in mild PH, when FAC does not change, the RV GLS begins to decrease (Li et al., 2013). Moreover, the correlation of RV GLS with the gold standard cardiac magnetic resonance-derived RV ejection fraction (RVEF) is better than the correlation of RV FAC and TAPSE with RVEF (Tong et al., 2018; Kavurt et al., 2019); a lower RV GLS may indicate a lower RVEF in notch (+) subjects. These findings are consistent with those of a previous study which reported that the presence of an RVOT notch was related to a reduced RV SV index in patients with PH (Arkles et al., 2011). Intraventricular RV dyssynchrony is an early sign of RV contractile dysfunction. In our previous study, we had found that notch (+) cases in RV dyssynchrony (−) subjects and RV dyssynchrony (+) subjects were comparable. However, in this study, we found that the RVOT notch was associated with higher RVSD4, which is the indicator of RVD. This inconsistency may be attributed to the different ascending modes, exposure times, and ratio of males and females. In the present 2-day period HA exposure study, the subjects with an RVOT notch are more prone to suffer intraventricular RV mechanical dyssynchrony (RVSD4 > 18.7 ms) compared with subjects without an RVOT notch (Badagliacca et al., 2015; Yang et al., 2020). In this study, higher RV dyssynchrony was accompanied by a lower RV GLS. A similar observation was reported in patients after hemodialysis (Unlu et al., 2019).

### Impact of Mid-Systolic DT on Cardiac Function

Previous studies reported that PH patients with mid-systolic DT < 120 ms had worse clinical outcomes compared with those subjects with mid-systolic DT > 120 ms (Takahama et al., 2017). In this study, the RVD base and RVD mid in mid-systolic DT < 120 ms group were larger than those in mid-systolic DT > 120 ms group. RV dilation is a sign of RV maladaptation in response to high PAP; however, the outcomes of subjects with mid-systolic DT < 120 ms upon HA exposure still need long-term follow-up studies to investigate.

Several limitations in this study should be noted. For ethical reasons, cardiac catheterization, the gold standard for the measurement of cardiac hemodynamics, was not performed in this study. As 30 females (30.3% of total subjects) were included in this study and the menstrual cycle in female participants may affect heart physiology, menstrual cycle information should be collected, and its association with RVOT notch needs to be investigated. Acute mountain sickness was reported to be associated with reduced LV function, whereas, as this study mainly focused on the association of RVOT notch with RV function, acute mountain sickness cases were not excluded. As the subjects in this study returned to low altitude within two months, the long-term association of an RVOT notch with RV function was not studied. It is not clear whether the subjects with RVOT notch are more likely to develop HA heart disease. Furthermore, the cardiopulmonary exercise test to investigate RV contractile reserves in subjects with an RVOT notch should be performed in future studies as the RV function in these subjects is maladapted to HA exposure. It would be valuable to see whether the RVOT notch would reverse to normal after notch (+) subjects returned to sea level and whether a lower exercise capacity could be observed in the notch (+) subjects at sea level via cardiopulmonary exercise test.

## Conclusion

Our study revealed that HA exposure-induced RVOT notch formation may be attributed to high PAP and low SpO_2_. The presence of an RVOT notch upon HA exposure was associated with impaired RV function as the tricuspid ICV and s’ did not increase, the RV deformation reduced, the RV-PA coupling deteriorated, and RV intraventricular dyssynchrony was observed. Therefore, an RVOT notch may be a potential echocardiographic sign representing impaired RV function at HA. However, whether the impaired RV function in subjects with RVOT notch upon HA exposure persists or is transient still needs further investigation.

## Data Availability Statement

The raw data supporting the conclusions of this article will be made available by the authors, without undue reservation.

## Ethics Statement

The studies involving human participants were reviewed and approved by the Clinical Research Ethics Board of Army Medical University. The patients/participants provided their written informed consent to participate in this study.

## Author Contributions

LH, FY, and CL contributed to the conception or design of the work. FY, CL, JK, YY, CH, CZ, RR, ZL, and JuY conducted the experiments. FY and CL performed the statistical analyses and drafted the manuscript. SY, SB, JiY, XD, JZ, and HT interpreted the results of the statistical analyses. LH critically revised the manuscript. All authors approved of the final version of the manuscript and agreed to be accountable for all aspects of the work.

## Conflict of Interest

The authors declare that the research was conducted in the absence of any commercial or financial relationships that could be construed as a potential conflict of interest.
