# Supplementary material for: The Association Between Notching of the Right Ventricular Outflow Tract Flow Velocity Doppler Envelope and Impaired Right Ventricular Function After Acute High-Altitude Exposure
Source: Front Physiol. 2021 Apr 1;12:639761. doi: 10.3389/fphys.2021.639761 (PMC8047424; doi:10.3389/fphys.2021.639761)
Supplement: Supplementary file 1 [file Table_1.DOCX]

**Supplemental Table S1. ICC analysis of intra- and interobserver variations**

| Variables | Intraobserver variation | | p value | Interobserver variation | | p value |
| --- | --- | --- | --- | --- | --- | --- |
|  | ICC | 95%CI |  | ICC | 95%CI |  |
| LV EDV, ml | 0.933 | 0.839-0.973 | <0.001 | 0.953 | 0.887-0.981 | <0.001 |
| LV ESV, ml | 0.972 | 0.930-0.989 | <0.001 | 0.978 | 0.945-0.991 | <0.001 |
| Mitral E, cm/s | 0.969 | 0.924-0.988 | <0.001 | 0.954 | 0.887-0.981 | <0.001 |
| Mitral A, cm/s | 0.961 | 0.903-0.984 | <0.001 | 0.953 | 0.884-0.981 | <0.001 |
| Mitral ICV septal, cm/s | 0.973 | 0.935-0.989 | <0.001 | 0.969 | 0.924-0.988 | <0.001 |
| Mitral ICV lateral, cm/s | 0.972 | 0.931-0.989 | <0.001 | 0.970 | 0.926-0.988 | <0.001 |
| Mitral s’ septal, cm/s | 0.925 | 0.822-0.969 | <0.001 | 0.934 | 0.842-0.973 | <0.001 |
| Mitral s’ lateral, cm/s | 0.947 | 0.872-0.979 | <0.001 | 0.936 | 0.845-0.974 | <0.001 |
| Mitral e’ septal, cm/s | 0.937 | 0.849-0.975 | <0.001 | 0.930 | 0.825-0.972 | <0.001 |
| Mitral e’ lateral, cm/s | 0.925 | 0.822-0.969 | <0.001 | 0.905 | 0.776-0.961 | <0.001 |
| LV GLS, % | 0.885 | 0.736-0.953 | <0.001 | 0.860 | 0.684-0.942 | <0.001 |
| LV GCS, % | 0.935 | 0.847-0.974 | <0.001 | 0.955 | 0.891-0.982 | <0.001 |
| TS-12SD, ms | 0.877 | 0.714-0.949 | <0.001 | 0.863 | 0.687-0.943 | <0.001 |
| TRV, cm/s | 0.956 | 0.894-0.982 | <0.001 | 0.952 | 0.885-0.981 | <0.001 |
| RV EDA, cm2 | 0.967 | 0.915-0.987 | <0.001 | 0.955 | 0.892-0.982 | <0.001 |
| RV ESA, cm2 | 0.977 | 0.944-0.991 | <0.001 | 0.970 | 0.927-0.988 | \| <0.001 \| \| --- \| |
| TAPSE,cm/s | 0.922 | 0.816-0.968 | <0.001 | 0.943 | 0.863-0.977 | <0.001 |
| Tricuspid E, cm/s | 0.938 | 0.851-0.975 | <0.001 | 0.942 | 0.859-0.977 | <0.001 |
| Tricuspid A, cm/s | 0.975 | 0.937-0.990 | <0.001 | 0.966 | 0.917-0.986 | <0.001 |
| Tricuspid ICV septal, cm/s | 0.925 | 0.920-0.970 | <0.001 | 0.881 | 0.728-0.951 | <0.001 |
| Tricuspid ICV lateral, cm/s | 0.985 | 0.962-0.994 | <0.001 | 0.980 | 0.952-0.992 | <0.001 |
| Tricuspid s’ septal, cm/s | 0.935 | 0.844-0.973 | <0.001 | 0.914 | 0.797-0.965 | <0.001 |
| Tricuspid s’ lateral, cm/s | 0.946 | 0.870-0.978 | <0.001 | 0.930 | 0.833-0.972 | <0.001 |
| Tricuspid e’ septal, cm/s | 0.937 | 0.849-0.975 | <0.001 | 0.957 | 0.896-0.983 | <0.001 |
| Tricuspid e’ lateral, cm/s | 0.968 | 0.920-0.987 | <0.001 | 0.972 | 0.933-0.989 | <0.001 |
| RV GLS, % | 0.908 | 0.783-0.963 | <0.001 | 0.895 | 0.756-0.957 | <0.001 |
| RVSD4, ms | 0.857 | 0.679-0.940 | <0.001 | 0.805 | 0.572-0.918 | <0.001 |

Abbreviations: ICC, intraclass correlation coefficient; LV, left ventricular; EDV, end-diastolic volume; ESV, end-systolic volume; E, early diastolic transmitral or transtricuspid flow velocity; A, late diastolic transmitral or transtricuspid flow velocity; ICV, peak velocity of isovolumic contraction period at mitral or tricuspid annulus; s’, mitral or tricuspid annulus systolic velocity; e’, mitral or tricuspid annulus early diastolic velocity; GLS, global longitude strain; GCS, global circumferential strain; TS-12SD, the standard deviation of time to peak systolic strain in 12 LV segments; TRV, tricuspid regurgitation velocity; RV, right ventricular; EDA, end-diastolic area; ESA, end-systolic area; TAPSE, tricuspid annulus plane systolic excursion; RVSD4, standard deviation of the time to peak systolic strain in the 4 mid-basal RV segments;

**Supplemental Table S2. Pulmonary haemodynamics and right ventricular parameters in the notch (+) subjects with mid systolic deceleration time < 120 ms or with mid systolic deceleration time > 120 ms at low altitude and high altitude**

| **Variables** | **Mid systolic DT > 120 ms (n=13)** | | |  | **Mid systolic DT < 120 ms (n=7)** | | |
| --- | --- | --- | --- | --- | --- | --- | --- |
|  | Low altitude | High altitude | P value |  | Low altitude | High altitude | P value |
| **Pulmonary haemodynamics** | | | |  |  | | |
| SPAP, mmHg | 21.7 ± 3.3 | 31.1 ± 3.2 | 0.375 |  | 20.6 ± 1.5 | 40.0 ± 6.5 | 0.063 |
| mPAP, mmHg | 15.2 ± 2.0 | 21.0 ± 1.9 | 0.375 |  | 14.6 ± 0.9 | 26.4 ± 4.0 | 0.063 |
| PVR, WU | 1.6 (1.3, 2.1) | 2.1 (1.2, 4.0) | 0.625 |  | 0.6 (0.4, 1.8) | 4.0 (1.7, 4.0) | 0.125 |
| AT_RVOT_, ms | 148.0 (136.2, 156.4) | 129.2 (101.5, 139.7) | **0.011** |  | 139.9 (125.5, 162.5) | 110.5 (95.10, 119.0) | 0.109 |
| ET_RVOT_, ms | 365.1 (338.7, 394.7) | 348.4 (329.9, 372.1) | 0.244 |  | 355.1 (326.2, 406.9) | 365.1 (329.7, 381.5) | 0.813 |
| AT_RVOT_/ET_RVOT_ | 0.39 (0.36, 0.44) | 0.35 (0.30, 0.41) | **0.015** |  | 0.38 (0.36, 0.42) | 0.29 (0.27, 0.37) | 0.078 |
| PCWP, mmHg | 10.0 (9.1, 10.7) | 9.2 (8.7, 10.0) | **0.043** |  | 10.3 (9.2, 10.8) | 8.8 (8.5, 9.7) | 0.109 |
| **Conventional Doppler echocardiography** | | | |  |  | |  |
| RVD base, cm | 3.3 (2.9, 3.6) | 3.2(3.1 3.7) | 0.435 |  | 3.2 (3.1, 3.8) | 3.7 (3.5, 4.2)**^*^** | 0.094 |
| RVD mid, cm | 2.9 (2.4, 3.9) | 3.2 (3.0, 3.6) | 0.673 |  | 2.5 (2.3, 3.0) | 3.9 (3.3, 4.3)**^*^** | **0.016** |
| RVEDA, cm^2^ | 19.4 ± 1.0 | 19.1 ± 0.90 | 0.907 |  | 20.0 ± 0.5 | 20.1 ± 1.3 | 0.999 |
| RVESA, cm^2^ | 10.2 ± 0.9 | 11.0 ± 0.6 | 0.197 |  | 10.9 ± 0.6 | 12.2 ± 1.1 | 0.297 |
| RV FAC, % | 47.8 ± 2.2 | 43.0 ± 1.2 | 0.106 |  | 45.7 ± 1.6 | 40.6 ± 2.0 | 0.156 |
| TAPSE, mm | 2.6 (2.4, 2.9) | 2.4 (2.2, 2.5) | **0.008** |  | 2.6 (2.4, 3.0) | 2.3 (1.9, 2.4) | 0.078 |
| TAPSE/SPAP | 1.1 (0.9, 1.0) | 1.0 (0.7, 1.0) | 0.375 |  | 1.3 (1.1, 1.5) | 0.7 (0.4, 1.0) | 0.125 |
| Tricuspid E/A ratio | 1.7 (1.3, 2.4) | 1.1 (0.9, 1.7) | **0.020** |  | 1.6 (1.4, 2.7) | 1.3 (1.0, 1.3) | **0.047** |
| **Pulse tissue Doppler imaging** | | | |  |  | |  |
| Tricuspid s’, cm/s | 11.3 (9.9, 13.2) | 11.3 (10.6, 12.4) | 0.732 |  | 11.6 (9.7, 11.75) | 11.0 (9.3, 11.8) | 0.999 |
| Tricuspid e’, cm/s | 13.2 (11.8, 15.8) | 12.8 (11.5, 14.5) | 0.213 |  | 14.0 (10.3, 16.4) | 14.1 (8.8, 18.5) | 0.844 |
| Tricuspid ICV, cm/s | 7.8 (7.6, 9.0) | 7.8 (6.4, 9.7) | 0.866 |  | 7.8 (6.7, 10.0) | 8.4 (7.4, 11.2) | 0.156 |
| **Speckle tracking imaging** | | | |  |  | | |
| RV GLS, % | -21.5 (-18.2, -23.3) | -18.7 (-15.5, -22.4) | 0.090 |  | -22.4 (-19.3, -23.7) | -18.1 (-16.0, -23.0) | 0.109 |
| 2DS RVFW apex, % | -24.0 (-21.0, -25.5) | -20.0 (-16.0, -24.0) | 0.061 |  | -26.0 (-21.0, -27.0) | -18.0 (-17.0, -24.0) | 0.063 |
| 2DS RVFW mid, % | -23.0 (-18.5, -25.0) | -20.0 (-15.0, -24.0) | 0.052 |  | -22.0 (-19.0, -26.0) | -20.00 (-16.0, -26.0) | 0.563 |
| 2DS RVFW base, % | -17.0 (-14.5, -22.0) | -14.0 (-11.5, -20.0) | 0.174 |  | -18.0 (-13.0, -21.0) | -14.00 (-14.0, -19.0) | 0.313 |
| RVSD4, ms | 6.5 ± 1.0 | 11.2 ± 1.5 | **0.012** |  | 4.6 ± 0.9 | 12.8 ± 4.2 | 0.375 |

SPAP, systolic pulmonary artery pressure; mPAP, mean pulmonary artery pressure; PVR, pulmonary vascular resistance; AT_RVOT_, acceleration time of RVOT; ET_RVOT_, ejection time of RVOT; PCWP, pulmonary capillary wedge pressure; RVD, right ventricular transverse diameter; FAC, fraction area change; 2DS RVFW, 2-dimensional strain of right ventricular free wall. Other abbreviations as in Supplemental Table S1.

**Supplemental Table S3. Logistic regression analysis of the correlated parameters with notch occurrence at high altitude**

| Variables | Univariate Analysis | |  | Multivariate Analysis | |
| --- | --- | --- | --- | --- | --- |
|  | p value | OR (95% CI) |  | p value | OR (95% CI) |
| Age | 0.126 | 1.05 (0.99, 1.11) |  | Not selected | |
| Sex | 0.294 | 1.73 (0.62, 4.80) |  | Not selected | |
| BMI | 0.643 | 1.05 (0.86, 1.28) |  | Not selected | |
| Smoking | **0.010** | 0.13 (0.03, 0.61) |  | —— | |
| HR | 0.168 | 1.03 (0.99, 1.07) |  | Not selected | |
| SpO_2_ | 0.065 | 0.88 (0.77, 1.01) |  | Not selected | |
| SBP | 0.062 | 1.03 (1.00, 1.06) |  | Not selected | |
| DBP | 0.172 | 1.03 (0.99, 1.06) |  | Not selected | |
| CO | 0.436 | 1.21 (0.75, 1.95) |  | Not selected | |
| LVEF | 0.980 | 1.00 (0.91, 1.10) |  | Not selected | |
| Ts12SD | 0.945 | 1.00 (0.95, 1.05) |  | Not selected | |
| LV GLS | 0.522 | 0.92 (0.72, 1.18) |  | Not selected | |
| LV GCS | 0.187 | 0.87 (0.71, 1.07) |  | Not selected | |
| Mitral E/A | **0.019** | 0.16 (0.04, 0.75) |  | —— | |
| Mitral ICV | 0.345 | 1.11 (0.89, 1.40) |  | Not selected | |
| Mitral s’ | 0.068 | 0.76 (0.57, 1.02) |  | Not selected | |
| Mitral e' | 0.050 | 0.82 (0.67, 1.00) |  | Not selected | |
| RVD base | 0.680 | 1.21 (0.50, 2.92) |  | Not selected | |
| RVD mid | 0.721 | 1.13 (0.59, 2.14) |  | Not selected | |
| RV EDA | 0.606 | 0.95 (0.80, 1.14) |  | Not selected | |
| RV ESA | 0.965 | 1.01 (0.77, 1.32) |  | Not selected | |
| RV FAC | 0.255 | 0.94 (0.85, 1.05) |  | Not selected | |
| TAPSE | 0.231 | 0.44 (0.11, 1.70) |  | Not selected | |
| Tricuspid E/A | **0.021** | 0.23 (0.07, 0.80) |  | **0.016** | 0.13 (0.03, 0.68) |
| Tricuspid ICV | 0.361 | 0.91 (0.73, 1.12) |  | Not selected | |
| Tricuspid s' | 0.281 | 0.83 (0.59, 1.16) |  | Not selected | |
| Tricuspid e' | 0.151 | 0.88 (0.74, 1.05) |  | Not selected | |
| RV GLS | **0.025** | 0.84 (0.72, 0.98) |  | **0.011** | 0.74 (0.60, 0.93) |
| RVSD4 | **0.004** | 1.14 (1.04, 1.24) |  | —— | |
| AT_RVOT_ | 0.965 | 1.00 (0.98, 1.02) |  | Not selected | |
| ET_RVOT_ | 0.349 | 0.99 (0.98, 1.01) |  | Not selected | |
| SPAP | **0.004** | 1.08 (1.03, 1.14) |  | **0.001** | 1.14 (1.05, 1.23) |
| PCWP | 0.644 | 1.08 (0.78, 1.49) |  | Not selected | |
| PVR | **0.007** | 1.74 (1.16, 2.59) |  | Not selected | |

OR, odds ratio; BMI, body mass index; HR, heart rate; SpO_2_, pulse oxygen saturation; SBP, systolic blood pressure; DBP, diastolic blood pressure; LVEF, left ventricular ejection fraction; CO, cardiac output; Other abbreviations as in Supplemental Table S1 and S2. Due to PVR is derived from SPAP, PVR has been excluded from the multivariate regression model.
